# Predicting Cardiovascular Risk Factors for Acute Leukemia Patients by Assessing Subclinical Atherosclerosis and Left Ventricular Function Before Chemotherapy

**DOI:** 10.3390/life15050704

**Published:** 2025-04-27

**Authors:** Anda Gabriela Militaru, Daniel Florin Lighezan, Anca Maria Cimpean, Elena Amaricai, Marius Militaru

**Affiliations:** 1 Department of Internal Medicine I, Medical Semiology I, “Victor Babes” University of Medicine and Pharmacy Timisoara, Municipal Emergency Hospital Timisoara, Eftimie Murgu Square No 2, 300041 Timisoara, Romania; militaru.anda@umft.ro (A.G.M.); dlighezan@umft.ro (D.F.L.); 2 Center of Advanced Research in Cardiology and Hemostasology, University of Medicine and Pharmacy “Victor Babes” Timisoara, E. Murgu Square, Nr. 2, 300041 Timisoara, Romania; marius.militaru@umft.ro; 3 Department of Microscopic Morphology/Histology, “Victor Babes” University of Medicine and Pharmacy Timisoara, Eftimie Murgu Square No 2, 300041 Timisoara, Romania; 4 Center of Expertise for Rare Vascular Disease in Children, Emergency Hospital for Children Louis Turcanu, 300011 Timisoara, Romania; 5 Center for Genomic Research, GENOMICA, “Victor Babes” University of Medicine and Pharmacy Timisoara, Eftimie Murgu Square No 2, 300041 Timisoara, Romania; 6 Research Center for Pharmaco-Toxicological Evaluation, Victor Babeș University of Medicine and Pharmacy, Eftimie Murgu Square No 2, 300041 Timisoara, Romania; 7 Department of Rehabilitation, Physical Medicine and Rheumatology, Research Center for Assessment of Human Motion, Functionality and Disability, “Victor Babes” University of Medicine and Pharmacy Timisoara, Eftimie Murgu Square No 2, 300041 Timisoara, Romania; amaricai.elena@umft.ro; 8 Department of Neuroscience, Neurology II, “Victor Babes” University of Medicine and Pharmacy Timisoara, Municipal Emergency Hospital Timisoara, Eftimie Murgu Square No 2, 300041 Timisoara, Romania

**Keywords:** leukemia, cardiovascular risk factors, left ventricle dysfunction, subclinical atherosclerosis, cardiotoxicity

## Abstract

Background: Subclinical atherosclerosis is a “silent” cardiovascular disease that can be devastating when combined with other illnesses. Its presence may affect therapy responses but can potentially worsen hematological malignancies due to most chemotherapy regimens’ cardiovascular adverse effects. Thus, cardiovascular risk factor (CVRF) assessment is required before chemotherapy. Unfortunately, this rarely happens. Aim: we aim to examine the impact of CVRFs on hemodynamic parameters of acute leukemia (AL) patients before chemotherapy. Methods: Overall, 45 AL patients and 26 controls were included. Intima-media thickness (IMT), ankle brachial index (ABI), pulse wave velocity (PWV), and functional cardiac parameters were used. CVRFs were found in 26 AL patients (36.6%), while 19 AL (26.8%) patients lacked CVRFs. CVRFs were also found in 26 controls (36.6%). Results: Left ventricular ejection fraction (LVEF) significantly decreased for patients with CVRFs (59.26 ± 5.62) compared to those without CVRFs (64.05 ± 7.43, *p* < 0.05). Hypertensive and diabetic patients had a significantly higher left IMT (mm) of 0.92 ± 0.01 compared to those without them (0.76 ± 0.03, *p* < 0.05). Patients with acute myeloid leukemia (AML) with CVRFs had a significantly higher PWV (m/s) of 8.4 ± 0.12 compared to those without CVRFs (6.87 ± 0.66) (*p* < 0.05). Conclusions: AL and cardiovascular risk factors interacted before chemotherapy. To decrease cardiotoxicity, AL patients need cardiovascular risk assessment. Subclinical atherosclerosis and echocardiography help chemotherapy patients to choose a treatment regimen, predict long-term outcomes, and predict cardiovascular issues.

## 1. Introduction

The effects of chemotherapy on heart function are well documented. These therapeutic regimens can have significant cardiovascular consequences. Cardiotoxicity can start early in chemotherapy; therefore, it is vital to recognize it [1,2]. Correcting these symptoms depends on their speed of detection. Heart failure (HF) symptoms are harder to treat if found late [2]. Special chemotherapy dosages may cure hematological diseases without increasing cardiotoxicity [3]. The induction stage of acute lymphoblastic leukemia (ALL) might cause 80% remission. It depends on age, risk factors, and comorbidities [4]. Cancer therapy-related cardiac dysfunction can occur as a result of doxorubicin treatment. Depending on cumulative dose, up to 9% of doxorubicin patients experience this [5].

AML incidence rises with age. In AML patients, anthracyclines are administered over three days (daunorubicin 45–90 mg/m^2^ per day, idarubicin 12 mg/m^2^ per day) with cytarabine (100–200 mg/m^2^ per day continuously for 7 days) (“713” regimen). Anthracyclines can cause cardiotoxicity, left ventricular ejection fraction (LVEF) impairment, HF, and arrhythmogenic right ventricular dysplasia (ARLVD). Higher cardiac comorbidities, CVRFs, and age may elevate ARLVD risk [6]. Arterial hypertension, chronic HF, CAD, PAD, and CKD are found in 60–80% of older persons [6]. Anthracycline-induced cardiotoxicity is classified as acute or chronic and defined as a >10% LVEF reduction to 50%. Several studies use different LVEF decline cutoff values [6]. Conventional assessment does not include decreased left fractional shortening (LVSF), global longitudinal strain (GLS), or diastolic dysfunction, which have been utilized to define anthracycline-related cardiotoxicity [6]. Another study indicated that just a minority of AML patients with ARLVD saw a cardiologist and received HF treatment [7]. To monitor ARLVD, LVEF should be monitored before, during, and after anthracycline therapy. GLS and CMRI can detect early heart injury and predict ARLVD. Basic assessment before therapy must include cardiac comorbidities and anthracycline-related left ventricular dysfunction risk factors [6]. Cardiotoxicity is higher in people with cardiac problems and CVRFs [2,8,9]. Because these patients must undergo cardiovascular examination before receiving any chemotherapeutic treatment, interprofessional teamwork is crucial [2,9]. Cardiotoxicity may increase with comorbidities such high blood pressure (BP), obesity, diabetes mellitus (DM), metabolic syndrome, or other cardiovascular diseases (CVDs) [10,11].

A medical history and physical examination should precede chemotherapy to stage the underlying illness and optimize heart function [11]. Cardiotoxicity risk is heavily influenced by risk profile [12]. To prevent CV injury, hematological malignancy patients must have their BP carefully assessed [13]. Early detection and continuous examination of AL patients with metabolic syndrome, obesity, dyslipidemia, high BP, glucose metabolism, and type 2 DM are needed to identify risk and implement preventative actions [14]. Lifestyle improvements and aging have increased CVD risk and cancer survival [15].

Echocardiography is essential for LV function evaluation before chemotherapy according to numerous clinical studies [2]. Tissue Doppler Imaging (TDI) and GLS are current echocardiographic approaches used to measure LV systolic and diastolic function. They can be utilized to detect CVRF-related changes in LV dysfunction before cancer treatment. Chemotherapy can exacerbate these alterations [16,17,18]. In neoplasia patients, diastolic dysfunction and right ventricle filling pressure must be assessed before and throughout chemotherapy [19].

According to the literature, anthracycline and radiation, especially in infancy, promote atherosclerotic alterations [15], but other chemotherapy medications can also cause these changes [20]. Young ALL patients who undergo chemotherapy are at risk for atherosclerosis. These individuals had elevated IL-6 and hs-CRP, which may indicate blood vessel endothelial lesions. After antineoplastic therapy, carotid artery IMT was statistically higher [21]. Any rise in IMT in cancer patients may indicate a risk of future cardiovascular or cerebrovascular problems. Increased IMT may worsen LV dysfunction [22,23]. Ankle brachial index (ABI) can be used to test for atherosclerosis and CVRFs. An increase in ABI is associated with a 2-fold increase in cardiovascular mortality, independent of gender, and leukemia patients may progress faster to atherosclerosis [24,25,26]. Lifestyle changes, greater life expectancy of cancer patients, and the potential of cardiotoxicity from chemotherapeutic medications or other therapeutic approaches have led to an emphasis on atherosclerotic disease. Long-term anticancer treatment may cause these problems [27]. As many diagnostic approaches as possible should be used to detect cardiac or vascular damage before it becomes symptomatic [28,29,30].

The European Society of Cardiology (ESC) *Cardio-Oncology Guide* advises AL patients to identify and rectify CVRFs by quitting smoking, exercising, and eating well. Predicting CVRF correction before and during chemotherapy is crucial to preventing cardiovascular problems [9]. Pre-existing decreased ventricular function increases the risk of ventricular dysfunction after chemotherapy. Pre-existing ventricular function parameters may increase the risk of ventricular dysfunction during chemotherapy [9,12,19]. The ESC cardio-oncology guidelines need a thorough understanding of pre-existing CVRFs and cardiovascular disease in oncologic patients [4].

Subclinical atherosclerosis was previously reported for pediatric leukemia survival patients as a side effect of chemotherapy on vascular structure and function [31]. Individuals who had survived leukemia exhibited diminished carotid distensibility and compliance, suggesting a heightened level of arterial stiffness in contrast to the control group. No notable disparities were observed in the assessments of carotid stiffness or endothelial dependent dilation (EDD) between survivors of solid tumors and central nervous system (CNS) tumors when juxtaposed with the control group. EDD exhibited a lower prevalence among leukemia survivors compared to the control group, whereas endothelial independent dilation (EID) was found to be elevated in survivors of CNS tumors relative to controls [31]. Sadurska et al. [21]. reported elevated levels of endothelial derived serum biomarkers in children receiving chemotherapy for acute lymphoblastic leukemia (ALL). Blood concentrations of the sICAM-1 adhesive molecule, the mean IMT values for right and left carotid arteries, and the concentration of hs-CRP were significantly higher in ALL patients after chemotherapy compared to control groups. The individuals who survived childhood ALL within the studied cohort exhibited heightened levels of specific novel biomarkers and elevated IMT values when juxtaposed with the control group, potentially substantiating the presence of endothelial damage in the vascular system. This research suggests that individuals who have undergone treatment for childhood malignancy face an elevated risk of developing atherosclerosis at an earlier stage in life. In young survivors of childhood acute lymphoblastic leukemia, cranial radiation therapy altered the distribution of adipose tissue and significantly influenced carotid intima-media thickness. The ratio of leptin to adiponectin, serving as a biomarker for abdominal obesity and metabolic syndrome, alongside diastolic blood pressure, also exerted an influence on carotid intima-media thickness, which is indicative of subclinical atherosclerosis [32].

Data about the impact of subclinical atherosclerosis on chemotherapy in adult patients with leukemia are very limited. With the exception of one previous paper from 2018 [29], no other direct studies of the relationship of subclinical atherosclerosis with leukemia treatment have been published.

As this is such a less studied subject, the primary aim of this study was to demonstrate the significance of identifying and managing cardiovascular risk factors in patients with acute leukemia to prevent potential cardiovascular complications arising from chemotherapy treatment. The study focused on assessing AL patients prior to the initiation of chemotherapy, rather than on monitoring these patients during chemotherapy treatment over time.

In this research, we sought to highlight the fact that AL patients with CVRFs need more cautious monitoring after therapy begins since CVRFs may increase the risk of cardiac injury. Thus, identifying high-risk individuals allows for greater surveillance, the use of cardioprotective medications, and targeted risk factor modification to decrease cardiotoxicity.

## 2. Materials and Methods

### 2.1. Patients’ Selection

This is a one-year prospective study that was developed in the Hematology and Internal Medicine Clinic. About 500 patients are admitted into the clinic yearly for various hematological malignancies and other blood disorders. From this patient pool, the AL patients were selected based on clinical and laboratory criteria, a diagnosis of AL according to the hematology guidelines and protocols, and an ability to meet the required study parameters without acute pathology decompensation that would make them unable to perform the required assessments. Patients with AL were diagnosed during study entry that month, with only those who were able to make all the required study assessments selected. This study assessed 71 participants, comprising 45 patients diagnosed with AL who were tested for cardiovascular risk factors prior to chemotherapy, and 26 controls without AL, who also presented with cardiovascular risk factors. The seventy-one patients participating in the study were admitted to the Hematology and Internal Medicine Clinic at Timisoara Municipal Emergency Clinic Hospital, Romania, throughout the years 2020 and 2021. Forty-five patients, diagnosed with acute leukemia (AML or ALL) and admitted to the Hematology Clinic, were assessed prior to the initiation of treatment.

**Figure 1:**
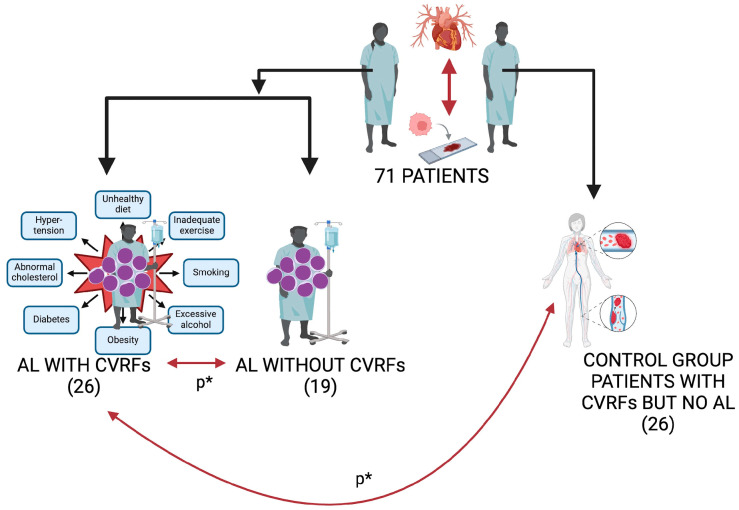
Patient groups and study design. AL: acute leukemia; CVRFs: cardiovascular risk factors. * *p* value corresponding to comparison of AL with CVRF versus AL without CVRF patients; * *p* value corresponding to comparison before chemotherapy treatment of AL and CVRF patients versus controls with CVRFs.

Twenty-six control patients without AL and with CVRFs were admitted to the Internal Medicine Clinic (Figure 1). The control group comprised AL-free patients with cardiovascular risk factors (CVRFs). This facilitated the comparison of the impact and dynamics of CVRFs on patients undergoing AL and chemotherapy with those without AL and chemotherapy.

Patients were older than 18 years, with a mean age of 53.28 ± 15.27 (19–79 years).

Among the 45 AL patients participating in the trial, 19 (42.23%) were without cardiovascular risk factors (CVRFs), while 26 (57.77%) had CVRFs. Among the 45 AL patients involved in the trial, 22 (48.9%) were diagnosed with AML, while 23 (51.1%) were diagnosed with ALL.

### 2.2. Inclusion and Exclusion Criteria

The inclusion criteria were as follows: patients diagnosed with acute leukemia (AML or ALL), with or without cardiovascular risk factors (CVRFs), without a prior history of cardiovascular or cerebrovascular disorders, and without previous chemotherapy treatment. The cardiovascular risk factors (CVRFs) were identified as the existence of hypertension, diabetes mellitus, dyslipidemia, obesity, smoking, and an age above 60 years. Control participants had cardiovascular risk factors, lacked a prior history of cardiovascular or cerebrovascular disorders, and had not undergone previous chemotherapy treatment. Clinical assessments, comprising medical histories and clinical examinations, were conducted for all patients. The registered parameters included BMI, HR, SBP, DBP, and paraclinical assessments. These were determined via blood tests, resting ECG, transthoracic echocardiography, and arterial indicators of subclinical atherosclerosis IMT in terms of ECD, ABI, and PWV. The echocardiographic evaluation adhered to the guidelines established by the American Society of Echocardiography (ASE) and the European Association of Cardiovascular Imaging (EACVI) concerning the identification of echocardiographic parameters that characterize systolic and diastolic function in these patients [19].

Patients with a clinical history of other hematological and non-hematological malignancies previously treated with any chemotherapy regimens and/radiotherapy were excluded from the present study.

### 2.3. Primary and Secondary Objectives Details

*Primary objective 1:* The objective of our study was to demonstrate that cardiovascular risk factors (CVRFs), defined as the presence of hypertension, diabetes mellitus, dyslipidemia, obesity, smoking, and an age over 60 years, are correlated with alterations in markers of subclinical atherosclerosis. This was assessed by measuring intima-media thickness (IMT) using Extracranial Doppler (ECD), ankle brachial index (ABI), pulse wave velocity (PWV), and modifications in left ventricular (LV) systolic and diastolic function through echocardiographic parameters, prior to chemotherapy in patients with AL. Patients with AL with cardiovascular risk factors were compared to patients with AL without cardiovascular risk factors.

*Primary objective 2:* The AL patients with cardiovascular risk factors were compared to controls (subjects without AL) who also had cardiovascular risk factors. A comparison with control patients possessing cardiovascular risk factors (CVRFs) was conducted to determine whether the existence of AL in patients with CVRFs altered echocardiographic parameters and subclinical atherosclerosis, in contrast to control patients with CVRFs who did not have AL and lacked related cardiovascular disease. The comparison between patients with AL and those with CVRFs, as well as individuals with CVRFs without AL, aimed to determine if the existence of AL in patients with CVRFs serves as a forerunner to the progression towards clinically evident cardiovascular disease.

The secondary objectives were to assess the types of acute leukemia (AML, ALL) prior to chemotherapy based on the presence or absence of cardiovascular risk factors (CVRFs) (secondary objective 1), to evaluate patients with acute leukemia (AML, ALL) according to sex with or without CVRFs (secondary objective 2), and to analyze patients over 60 years of age with or without CVRFs (secondary objective 3) in relation to biological, hemodynamic, vascular, and echocardiographic parameters.

ECD, ABI, PWV, and echocardiography were performed before chemotherapy. Before chemotherapy, SBP, DBP, HR, BMI, LV systolic and diastolic function, GLS, and subclinical atherosclerotic markers were assessed and associated with CVRFs.

The *European Cardio-Oncology Guide* intended to define cardiotoxicity precisely. CTRCD, defined by the ASE and EACVI, is a decrease in LVEF of >10% from baseline or <53% (normal reference value for 2D echocardiography ultrasound) due to chemotherapeutic agent cardiotoxicity [8,31]. The usual LVEF range is 53–73% [31]. Multiple cardiac imaging should confirm LVEF. LVEF echocardiography should be performed before treatment and 2–3 weeks afterward. Even if echocardiography LVEF is low, the patient may not have HF symptoms. Clinically, LVEF decreases can be symptomatic or asymptomatic, and echocardiographically reversible or irreversible. Reversible means LVEF returns within 5% of the initial value, while irreversible means LVEF improves by <10% compared to the lower limit and remains >5% below the initial value [31].

The research examines a crucial cardio-oncology topic: how CVRFs affect cardiotoxicity. High-cardiovascular-risk patients are in danger of cardiotoxicity, according to current research [12]. Patients in the research received 7 days of cytarabine and 3 days of daunorubicin in a program of periodic treatment based on clinical progression. Daunorubicin, an anthracycline, treats AL well. Therapeutic dosages were established using European Society of Hematology AL treatment standards. No doses exceeded the safe limit. To avoid cardiotoxicity, the *European Cardio-Oncology Guide* recommends a maximum therapeutic dose of 700 mg/m³ for anthracyclines in chemotherapeutic treatment. Above this dosage, LV systolic function may be impaired [2]. Chemotherapy can cause cardiotoxicity for months or years after treatment [2].

### 2.4. Parameters of Subclinical Atherosclerosis

#### 2.4.1. Carotid Intima-Media Thickness (IMT)

For the investigation of the vascular parameter IMT, we utilized a General Electric Vivid E9 echocardiograph equipped with a 9 MHz linear array transducer. We assessed 10 IMT measures for each patient in the distal segment of both common carotid arteries, approximately 1 cm from the carotid bulb, and utilized the mean of these results for each patient. The assessment of arterial stiffness was conducted utilizing a device known as the Medexpertarteriograph™ TL2 (Medexpert Ltd., Budapest, Hungary). This analytical procedure involved temporarily occluding the brachial artery using a strain gauge. Normal reference values for IMT were judged to be less than 0.99 mm [21].

#### 2.4.2. Aortic Pulse Wave Velocity (PWV) or Arterial Stiffness

PWV is crucial for cardiovascular event prediction. PWV is a non-invasive, reproducible cardiovascular risk calculation tool that can be used in clinical assessments [32]. It is necessary to measure the distance from the pubic bone to the overflow crest and transit time (RT/2). After wireless transmission, a computer processes the data. The surgery must be performed in a calm room where the patient is comfortable to avoid errors. One examiner conducted this maneuver before treatment in all AL patients. Normal reference values for PWV were < 10–12 m/s [33].

#### 2.4.3. The Ankle-Brachial Index (ABI)

ABI was performed on all AL patients before chemotherapy to detect peripheral vascular impairment, as several had cardiovascular risk factors. SonoTrax Vascular Doppler measured peripheral arterial flow. Other methods were used to measure SBP in the left and right upper limbs. In both lower limbs, the first Doppler signal and systolic arterial blood pressure were reduced in the pedios and posterior tibial arteries. ABI was determined using the BP ratio of the lower to upper limbs. The standard reference value for ABI is <0.9 [34].

### 2.5. Statistical Analysis

Analysis was conducted with the IBM SPSS Statistics program software for Windows, version 20.0. The results are presented as counts (percentages) for categorical data and as mean value ± standard deviation for continuous data. An analysis of blood tests, BMI, heart rate, blood pressure, vascular markers, and left ventricular function markers was conducted with independent T-tests among AL patients. We compared cardiovascular risk factors (CVRFs) in relation to AL patients without CVRFs, as well as between AL patients with CVRFs and control patients with CVRFs. Sex, age </> 60 years old, and patients having acute ALL versus acute ALM were also considered in the analysis. In addition, the Chi-squared test was used to examine differences between categorical variables. A *p* value of less than 0.05 was deemed statistically significant.

## 3. Results

### 3.1. Demographic Date in AL Patients with/Without CVRFs, and Control Patients with CVRFs

The participants in our study had a mean age of 53.28 ± 15.27 years (ranging from 19 to 79 years). Of the total, 33 (46.5%) were female and 38 (53.5%) were male. Out of 71 patients, 45 (63.4%) had AL; 26 (36.6%) had CVRFs and 19 (26.8%) did not; 26 (36.6%) were control patients with CVRFs. Among 45 AL patients, 26 individuals with AL (16 males, 61.5%; 10 women, 38.5%) presented with cardiovascular risk factors, with a mean age of 54.85 ± 16.63 years. Conversely, the 19 AL patients (10 men, 52.6%; 9 women, 47.4%) without cardiovascular risk factors had a mean age of 44.00 ± 18.75 years (*p* = 0.047). Among 26 control patients (36.6%), 12 (46.2%) were men and 14 (53.8%) were women with cardiovascular risk factors, with a mean age of 58.50 ± 5.21 years. Among the patients participating in the study, 38 (53.5%) resided in urban areas, while 33 (46.5%) were from rural areas (Table 1).

Out of the total number of patients included in our study, 52 (73.2%) were patients with CVRFs, with 26 (36.6%) in the AL with CVRF group of patients and 26 (36.6%) in the control with CVRF group of patients.

Among 26 AL patients with cardiovascular risk factors (CVRFs), 19 (73.1%) were hypertensive, 6 (23.2%) had diabetes mellitus, 4 (15.4%) had dyslipidemia, 2 (7.7%) were obese, and 13 (50%) were smokers. Among 26 control patients with cardiovascular risk factors (CVRFs), 16 (61.5%) were hypertensive, 7 (26.9%) had diabetes mellitus, 7 (26.9%) had dyslipidemia, 3 (11.5%) were obese, and 10 (38.5%) were smokers.

### 3.2. Biological Parameters in AL Patients with/Without CVRFs and Control Patients with CVRFs Before Chemotherapy Treatment

Our study observed a statistically significant reduction in Alanine aminotransferase (ALT) levels, measuring 35.11 ± 25.69 U/L in patients with AL and cardiovascular risk factors (CVRFs) compared to 52.89 ± 32.49 U/L in patients with AL without CVRFs prior to therapy initiation (*p* < 0.05). Potassium (K) levels were significantly reduced to 4.05 ± 0.60 nmol/L in patients with acute leukemia (AL) and cardiovascular risk factors (CVRFs) compared to 4.54 ± 0.73 nmol/L in patients with AL without CVRFs prior to the initiation of chemotherapy (*p* < 0.05). We observed statistically significant decreases in Hb (g/dL)—10.77 ± 2.48—and Ht (%)—33.03 ± 7.37—in patients with AL with CVRFs, compared to Hb values of (g/dL) 13.04 ± 1.69 and Ht (%) 39.72 ± 4.52 in control patients with CVRFs, prior to the initiation of treatment (*p* < 0.001). The alterations seen in other biological markers failed to reach statistical significance when comparing patients with AL and CVRFs to those without CVRFs or to control patients. The lipid profile parameters decreased in patients with cardiovascular risk factors (CVRFs) compared to those without, likely due to their prior knowledge of CVRFs and subsequent statin therapy before the onset of AL symptoms.

### 3.3. Hemodynamic and Subclinical Atherosclerosis Parameters in AL Patients with/Without CVRFs and in Control Patients Before Chemotherapy Treatment

Statistically significant changes were noted in hemodynamic parameters related to BMI (kg/m²), which decreased from 27.25 ± 3.64 in patients with cardiovascular risk factors (CVRFs) to 23.09 ± 3.67 in patients without CVRFs (*p* < 0.001). Systolic blood pressure (SBP) was lower in AL patients with CVRFs, at 126.53 ± 10.84 compared to 144.42 ± 20.75 in control patients with CVRFs (*p* < 0.001). Diastolic blood pressure (DBP) also decreased from 84.42 ± 12.75 in control patients with CVRFs to 76.92 ± 11.66 in AL patients with CVRFs (*p* < 0.05). However, heart rate and blood pressure variations did not show statistical significance between AL patients with CVRFs and those without CVRFs. IMT demonstrated a statistically significant increase in AL patients with CVRFs compared to control patients with CVRFs, particularly at the right carotid artery level (*p* < 0.05). IMT also increased at the left level of the carotid artery, while ABI decreased bilaterally and PWV increased in patients with AL and CVRFs, although these changes did not achieve statistical significance. Table 2 provides a detailed account of the hemodynamic parameters.

### 3.4. Echocardiography Parameters in AL Patients with/Without CVRFs and in Control Patients Before Chemotherapy Treatment

Left ventricular end-diastolic diameter (LVEDD), LVESD, LVEDV, and interventricular septum (IVS) were slightly increased, and fractional shortening (FS) and Mitral annular plane systolic excursion (MAPSE) were slightly decreased, in CVRF patients compared to those without CVRFs. Patients with AL and CVRFs had a significant increase in left ventricular posterior wall (LVPW) (10.15 ± 1.12 mm vs. 9.00 ± 1.20 mm) and left ventricular end systolic volume (LVESV) (31.30 ± 12.97 mL vs. 24.63 ± 6.28 mL, *p* < 0.05). Patients with AL and CVRFs had significantly lower LVEF (%) compared to AL patients without CVRFs (*p* < 0.05), and marginally lower values compared to control patients with CVRFs. Table 3 shows LV systolic and diastolic echocardiographic characteristics.

### 3.5. Speckle Tracking Assessment Through Global Longitudinal Strain (GLS) Parameters in AL Patients with/Without CVRFs and in Control Patients with CVRFs Before Chemotherapy Treatment

GLS, quantified through Speckle tracking imaging in apical 2 chambers and using the apical long axis, was slightly decreased in AL patients with CVRFs comparing to AL patients without CVRFs.

### 3.6. Biological, Hemodynamic, Vascular and Echocardiography Parameters in AML and ALL Patients with/Without CVRFs Before Chemotherapy Treatment

The study comprised 45 AL patients, 22 (48.9%) with AML and 23 (51.1%) with ALL. A lower left ventricular ejection fraction (LVEF) was seen in AML patients (58.52 ± 5.60) compared to ALL patients (64.18 ± 6.84) (*p* < 0.05). S’ (cm/s) was significantly lower in ALL patients (11.90 ± 3.31) compared to AML patients (13.90 ± 2.01; *p* < 0.05). GLS-A2C (%) significantly decreased in ALL patients, with a drop of −25.11 ± 3.44 compared to one of −27.15 ± 2.49 in AML patients (*p* < 0.05). Acute lymphoblastic leukemia (ALL) patients had lower hemoglobin, hematocrit, ALT, and AST levels than AML patients, but had higher glycemia levels (*p* < 0.05).

Overall, 73% of 23 ALL patients had cardiovascular risk factors. Patients with cardiovascular risk factors had significantly higher LVEDD, LVESD, and BMI values than those without (*p* < 0.05). Patients with cardiovascular risk factors (CVRFs) had a significant decrease in GLS-LAX (%), at −25.73 ± 3.74, compared to the decrease of −28.86 ± 1.38 in those without CVRFs (*p* < 0.05). Of 22 AML patients, 9 had cardiovascular risk factors. Patients with AML and cardiovascular risk factors (CVRFs) exhibited a significantly lower left ventricular ejection fraction (LVEF) (61.00 ± 7.05) compared to those AML patients without CVRFs (66.38 ± 6.00). A’ was significantly lower and LVPW was higher in cardiovascular risk factor patients compared to those without CVRFs (*p* < 0.05). LVEF (%) was statistically lower in patients with AL and CVRFs. (*p* = 0.018).

Hypertension significantly reduced left ventricular ejection fraction (LVEF) in 22 AML patients (58.42 ± 4.82) compared to non-hypertensive patients (66.86 ± 6.01, *p* < 0.05). A’ was significantly lower in patients with hypertension, smokers, patients with diabetes, and patients with dyslipidemia compared to those without risk factors (*p* < 0.05). Dyslipidemia increased LVPW, LVEDV, MAPSE, E’/A’, and PWV. Patients with dyslipidemia had substantially lower GLS-A2C (%) (*p* < 0.05) and GLS-AVG (%) (−22.80 ± 0.05) compared to those without dyslipidemia (−27.83 ± 1.60) (*p* < 0.05). Compared to non-smokers, smokers had substantial increases in E/A, A’, and E’ (*p* < 0.05). D-Dimer and PWV readings were significantly greater in smokers compared to non-smokers (*p* < 0.05). Smokers exhibited significantly lower GLS-A2C, A3C, and AVG levels (−25.23 ± 1.71 vs. −28.59 ± 1.13, *p* < 0.05).

Subclinical atherosclerosis indicators were lower in 23 acute lymphoblastic leukemia (ALL) patients with CVRFs than in those without. Patients with hypertension and diabetes exhibited considerably larger intima-media thickness (IMT) than those without hypertension or diabetes (*p* < 0.05). Non-smokers had a significantly higher right ankle brachial index (ABI) (1.16 ± 0.08) compared to smokers (1.03 ± 0.05) (*p* < 0.05). GLS-A2C decreased significantly in non-smokers (−26.49 ± 1.95) vs. (−23.60 ± 4.13) in smokers (*p* < 0.05). Patients with diabetes and hypertension exhibited higher E, LVEDD, and LVEDS and lower GLS-A3C compared to those without these cardiovascular risk factors (*p* < 0.05). Patients with dyslipidemia had significantly lower MAPSE scores (*p* < 0.05) compared to those without dyslipidemia. FS (%) was significantly lower in hypertension patients (35.58 ± 13.78) compared to non-hypertensive people (46.47 ± 9.13, *p* < 0.05). Smokers showed a significant increase in E’ and LDL cholesterol compared to non-smokers (*p* < 0.05).

### 3.7. Biological, Hemodynamic, Vascular and Echocardiography Parameters Regarding Sex, with/Without CVRFs in AL Patients Before Chemotherapy Treatment

Among the 45 AL patients enrolled in the study, 19 were women (10 with CVRFs and 9 without) and 26 were men (16 with CVRFs and 10 without). LVPW and IVS were significantly elevated in female patients with cardiovascular risk factors compared to those without (*p* < 0.05). GLS-A3C% was significantly lower in female patients without cardiovascular risk factors (CVRFs), measuring −28.68 ± 1.28. compared to −26.37 ± 2.90 in those with CVRFs (*p* < 0.05). The left ventricular ejection fraction (LVEF) was significantly lower in male patients with cardiovascular risk factors (CVRFs), measuring 60.06 ± 4.61, compared to 66.00 ± 5.20 in male patients without CVRFs (*p* < 0.05). In male patients with cardiovascular risk factors (CVRFs), LVESV, A’, and TG were significantly lower, while BMI, glycemia, and urea were significantly higher, compared to male patients without CVRFs (*p* < 0.05).

Among the 23 patients with ALL, there were 10 females and 13 males. In the group of 22 patients with AML, 9 were female and 13 were male. The left ventricular ejection fraction (LVEF) was significantly lower in female patients, recorded at 55.40 ± 4.03, compared to male patients with acute lymphoblastic leukemia (ALL), who had an LVEF of 60.92 ± 5.57 (*p* < 0.05). In female patients with ALL, LVEDV and E/A were significantly lower compared to male patients (*p* < 0.05). E and E/A levels were significantly lower in female patients compared to male patients with AML (*p* < 0.05).

### 3.8. Biological, Hemodynamic, Vascular and Echocardiography Parameters Regarding Age </>60 Years Old, with/Without CVRFs in AL (AML or ALL) Patients Before Chemotherapy Treatment

The trial included 45 acute leukemia patients, 28 (62.2%) of whom were under 60 (12 with acute myeloid leukemia and 16 with acute lymphoblastic leukemia) and 17 (37.8%) were over 60 (10 patients with AML and 7 with ALL).

In ALL patients over 60 years old, diastolic blood pressure (DBP) was considerably higher (82.11 ± 6.49 mmHg) than in these patients under 60 years old (73.03 ± 9.93 mmHg). Additionally, the older cohort had a substantially lower left ventricular ejection fraction (LVEF) (58.41 ± 6.49%) compared to the younger group (63.03 ± 6.48%) (*p* < 0.05). Additional systolic and diastolic echocardiographic parameters showed an increase in wave A (m/s) and E/A, while wave E’ (cm/s) and E’/A’ decreased significantly in patients over 60 years old compared to those under 60 (*p* < 0.05). Patients over 60 years old had significantly lower hemoglobin (g/dL) and hematocrit (%) compared to those under 60 years old (*p* < 0.05), while other biological, echocardiographic, vascular, or hemodynamic parameters did not change significantly with age in AL patients. Patients over 60 years old with AML had a significantly higher heart rate (b/min) and wave A (m/s), but lower wave S’, E’, E’/A’, GLS-A2C, and GLS-AVG compared to those under 60 years old (*p* < 0.05). Acute lymphoblastic leukemia (ALL) patients over 60 years old had statistically significantly higher SBP, DBP, D-Dimer levels (ng/mL), wave A velocity (m/s), and E/E’ ratios. Patients over 60 years old had significantly lower left ventricular ejection fraction (LVEF) and E’ wave velocity compared to those under 60 years old (*p* < 0.05).

This study comprised 26 patients with CVRFs and acute leukemia (AL), 15 of whom were under 60, with 11 over 60. Of the 19 AL patients without CVRFs, 13 were under 60 and 6 were over 60. Elderly AL and CVRF patients demonstrated statistically significant increases in SBP, DBP, D-Dimer, and A wave compared to younger individuals. Individuals over 60 years old had significantly lower hemoglobin, hematocrit, left ventricular ejection percent, S’ and E’ waves, and global longitudinal strain-A2C compared to those under 60 years old (*p* < 0.05). The remaining research parameters did not change statistically based on AL type (AML or ALL), CVRFs, or age over 60. In our study, patients over 60 displayed potential for changes in biochemical, hemodynamic, vascular, or echocardiographic characteristics.

## 4. Discussion

Due to molecular and immunological changes, leukemia is deadly. Chronic illnesses and age at treatment initiation affect therapy outcomes in this population of patients. These factors affect chemotherapy cardiotoxicity [2].

In our 71-person study, 45 (63.4%) had AL (AML or ALL) and 26 (36.6%) had CVRFs. The 45 AL patients included 26 (36.6%) CVRFs and 19 (26.8%) without them. All 45 AL patients were included in this study, regardless of whether they had CVRFs or not, and underwent their first cardiac exam before treatment. Before starting potentially CV-harmful cancer drugs, the ESC Heart Failure Association (HFA) and International Cardio-Oncology Society (ICOS) convened a workshop to assess baseline CV risk in cancer patients This study group thinks risk is ongoing and that many CVRFs can work together in cancer patients. Before therapy, all oncology patients undergo CV risk stratification. The cancer patient should understand CVD risk and participate in therapy and CV toxicity decisions.

Factors impacting CVRFs and LVEF include initial LVEF <50%; medium LVEF 50–54%; age ≥80 years; average age 65–79 years; hypertension; diabetes; and smoking history. VEGF or its inhibitors’ baseline CV risk categorization includes other CVRFs and dyslipidemia as medium-risk components. Future cardiotoxicity risk for each risk category relies on the CVRFs present; severity; and cancer treatment. This risk can be categorized as low (<2%), medium (2–9%), high (10–19%), and very high (20%+).

Cardio-oncology or CV monitoring may help intermediate-risk cancer patients. A cardio-oncology or cardiology service evaluates high- and very-high-risk patients to optimize pre-existing CVD and modifiable CVRF care and provide a monitoring plan throughout cancer therapy [12]. A customized cancer care strategy should include early CV risk assessment according to the Cardiology and Oncology Society [12]. Immediately identifying high-risk cancer patients allows for safe therapy and early cardiovascular disease detection [12]. Knowledge of patient risk factors impacts prognosis. All pre-chemotherapy studies reduce cardiotoxicity and improve survival [12]. Many studies have shown that cardiovascular or cerebrovascular disease risk factors and pre-existing diseases exacerbate cardiotoxicity; hence, chemotherapy patients must be informed [2,8].

Overall, 1–5% of cancer survivors develop CTRCD, and 20% have asymptomatic LV function decrease. Trastuzumab/pertuzumab and anthracyclines caused the earliest cardiac damage. LVEF fell 0.29% (*p* = 0.009) annually for 20 years following breast cancer diagnosis. The study advised CVRF and echocardiographic surveillance for years following chemotherapy to reduce cardiotoxic risk.

The study found older AML patients to be at risk of ARLVD. ARLVD risk stratification, utilizing image-based baseline cardiac function tests and other CVRF and symptom detection approaches, is advised. Although used judiciously, anthracyclines can induce leukemia. CVRF diagnosis and heart disease risk assessment affect treatment outcomes [6].

Lancelotti and colleagues found 29.2% hypertension and 6.5% diabetes rates in 783 oncology patients. Dyslipidemia was present in 24.3% of patients, with smoking found in 13.4%. This study found a 0.5% rate of acute heart failure (HF) and a 6.3% of rate pf cardiovascular pathogen patients before treatment [8]. Prior hypertension increased the incidence of post-chemotherapy cardiotoxicity [8,35,36].

In a 5-year cardio-oncology clinic trial, 1324 cancer patients at moderate/high risk for CV toxicity (reported severe cardiotoxicity (CTox) ≥ 2%) were examined to identify and modulate CVRFs. Echocardiographic parameters were measured before cancer treatment and at 3 weeks, 3 months, 6 months, 1 year, 1.5 years, and 2 years.

In this study, 13.9%, 9.8%, and 10.3% had hypertension, DM, and hypercholesterolemia. Systemic coronary risk (SCORE) categories were defined as follows: low to intermediate (<5%), moderate (5–10%), and very high (≥10%). The worst follow-up myocardial injury/dysfunction determined CTox severity. This classification classifies severe CTox as clinical HF or asymptomatic LVEF ≤ 40. CTox and all-cause mortality were substantially increased in SCORE 5–9 and HR 4.90 (95% CI 2.44–9.82) was increased for SCORE ≥ 10 compared to SCORE 0–4. This study found high baseline and follow-up CVRF prevalence and incidence. Chemotherapy exacerbated DM and cholesterol. Cancer patients should use SCORE for initial CV risk assessment because it was used to estimate CTox risk throughout follow-up [37].

Patients need early cardiotoxicity identification to increase protective mechanisms and avoid chemotherapy cessation [38]. AL patients with CVRFs had a 5% lower echocardiographic LVEF before therapy. CVRFs may reduce LVEF following chemotherapy, increasing cardiotoxicity risk; hence, patients require special care and echocardiograms before treatment. Later echocardiograms will show if these patients’ LVEF will decrease reversibly or irreversibly and if AL patients with CVRFs are more affected. The LVEF of AL patients with CVRFs was 5% lower before treatment, according to our study.

To predict trastuzumab-mediated cardiotoxicity, 118 HER2-positive early-stage invasive breast cancer patients’ baseline LV diastolic function was assessed. The cumulative epirubicin dose and baseline TPFR >180 ms independently predicted TMC in logistic analysis. Pre-treatment LV diastolic dysfunction predicts trastuzumab cardiotoxicity. Diastolic function may improve CV risk assessment before trastuzumab chemotherapy in cancer patients [39].

We found that AL patients with CVRFs had a lower LVEF upon first assessment than those without, making this crucial. In our study, patients without CVRFs and AL had a significantly greater LVEF (%) of 64.05 ± 7.43 compared to those with CVRFs and AL (59.26 ± 5.62).

Patients with AL had an average age of 50.27 ± 18.17, while those with AL and CVRFs had a mean age of 54.85 ± 16.63. CVRF patients were smokers, hypertensive, diabetic, or obese. Aging, AL, and chemotherapy may increase CVRFs, leading to cardiotoxicity and vascular damage. Patients over 60 had higher DBP (mmHg) and lower Hb, Ht, and LVEF values compared to those under 60 (*p* < 0.05). AL and CVRF patients under 60 years old had higher SBP, DBP, D-Dimer, and wave A values, whereas those over 60 had lower Hb, Ht, LVEF, S’wave, E’wave, and GLS-A2C values (*p* < 0.05).

Many cardiotoxicity changes were observed in elderly patients in field papers. Ssessment for CVRFs and cardiovascular disease before treatment may change this. Hypertensive, diabetic HF patients are more prone to experience cardiotoxicity after chemotherapy and radiotherapy [2].

Anthracycline cardiotoxicity causes LV dysfunction. Elderly people may have more CVRFs and cardiac comorbidities, worsening anthracycline-induced LV dysfunction [30]. Over 60% of AML patients are 60+. Several risk factors exist. The main risk factors for LV dysfunction are anthracycline dosage, female gender, age > 65 or < 18 years, renal failure, concurrent irradiation, alkylating agent chemotherapy, pre-existing cardiac disease, CVRFs such hypertension, and genetic factors [6]. Out of 26 AL/CVRF patients (mean age: 54.85 ± 16.63), 73.1% had hypertension, 50% smoked, 23.2% had diabetes, and 15.4% had dyslipidemia. Aging, CVRFs, and anthracycline may simplify chemotherapy-induced LV failure. MAPSE measures global and longitudinal LV systolic strain. MAPSE was considerably lower in dyslipidemia patients with ALL compared to those without dyslipidemia (*p* < 0.05).

Speckle tracking GLS in all patients before medication found heart illnesses or CVRFs that may cause such symptoms. This echocardiography showd cycle-related cardiac distortion. Deformation can be measured longitudinally, radially, and circumferentially [40,41]. An average was calculated from LV longitudinal plane deformation in all segments. If cytostatic medication affects the GLS, we must treat cardiovascular issues before chemotherapy, which may damage the heart. Numerous investigations link CVRFs and pre-existing cardiovascular disease to cardiotoxicity [2,41,42,43]. A 15% GLS drop from baseline indicates long-term LV dysfunction [24,41,42,43,44,45].

LV systolic dysfunction, cardiac mortality, or symptomatic HF are predicted by baseline GLS before anthracycline therapy in retrospective hematologic malignancies. GLS is a well-established and important tool for predicting subsequent cancer therapy-related cardiac dysfunction (CTRD) (typically defined as a decrease in LVEF <50 or 53% with ≥5% absolute reduction in symptomatic patients or ≥10% in asymptomatic patients during anthracycline therapy, even in patients without risk factors for CTRD). In a cohort of patients with cancer and a normal baseline LVEF, the aim of a study was to investigate whether the baseline assessment of GLS before anthracycline treatment is predictive for CTRD. Baseline GLS or a decrease in baseline GLS ≥- 18% was predictive of CTRD along with a history of tobacco use, SBP, pre-chemotherapy, and the cumulative anthracycline dose. These data support the implementation of echocardiography as a baseline protocol in practice cardio-oncology for the identification and monitoring of patients at increased risk of CTRD. Baseline GLS has predictive value in identifying patients at low to moderate risk before treatment with anthracycline CTRD.

Speckle tracking echocardiography can outline the CV risk of these patients, along with that demonstrated by the knowledge of pre-treatment comorbidities and CVRFs that increase the risk of cardiotoxicity in cancer therapy [46]. In our study, we noted that patients diagnosed with AL and who were identified as having dyslipidemia, as an example of a CVRF, had GLS-A2C (%) and GLS-AVG (%) values that were statistically significantly lower compared to patients without dyslipidemia (*p* < 0.05).

Age and CVRFs can modify systolic and diastolic features; therefore, periodic echocardiography exams after chemotherapy can show heart function, hematological disease progression, and cytostatic treatment.

Patients taking cardiotoxic drugs should under systolic and LV diastolic tests [41]. Diastolic characteristics do not imply cardiotoxicity. We found that CVRFs and aging alter diastolic parameters.

Some researchers studied chemotherapy-induced vascular wall alterations. Unlike atherosclerotic changes, 4D flow wave speed before or after chemotherapy and phase contrast CMR can measure arterial stiffness [41]. Regarding pulse wave speed or flow-mediated vascular dilation, vascular lesions in the studied area relate to endothelial dysfunction or vasa-vasorum [41]. Atheroma plaque and arterial wall thickness are assessed by CMR [41].

Chronic myeloid leukemia patients treated with nilotinib or ponatinib may develop severe lower extremity atherosclerotic and non-atherosclerotic PAD while not having cardiovascular disease risk factors [2].

PWV and carotid IMT indicate subclinical atherosclerosis and can cause target organ, cerebrovascular, and cardiovascular diseases [47].

PWV carotid-femoral, the “gold standard” for arterial stiffness measurement, predicts long-term cardiovascular events in diabetes, hypertension, and coronary artery disease patients [31,48,49]. Cardiovascular mortality is predicted well by PWV. One PWV study predicted long-term cardiovascular disease [50,51].

This current investigation found alterations in parameters like IMT in persons with vascular, cerebrovascular, cardiac pathology, CVD risk factors, and acute leukemia. Changes in IMT may indicate long-term cerebrovascular occurrences in these patients. Acute leukemia patients with cardiovascular risk factors (hypertension, diabetes) have higher IMT values.

In a study conducted on 190 patients with very high cardiovascular risk profiles, divided into two groups of patients (103 had chronic heart failure (CHF) (group A) and 87 did not (group B), the values of IMT were statistically significantly higher in patients from group A compared to those in patients from group B (*p* < 0.05). LVEF was statistically significantly lower (55.91 ± 7.37%) in patients from group A compared to those from group B (59.14 ± 6%) (*p* = 0.001). Cacho-Diaz B and colleagues observed that cancer is a major risk factor for embolic strokes, and individuals with comorbidities, a more critical clinical state, or cardiovascular risk factors have a worse prognosis.

Some studies say ABI can detect atherosclerosis or cardiovascular risk. Their investigation found that it predicts cardiovascular mortality and other risk variables [52,53]. A low ABI raises cardiovascular mortality regardless of gender [52].

Another ABI study indicated that hypertensive patients with atrial fibrillation (AF) had lower values than those without AF but no peripheral artery disease (PAD) [54].

Our investigation found that AL patients with CVRFs have lower baseline LVEF and greater atherosclerosis indices, which may enhance cardiotoxicity risk.

Low LVEF and a larger LV may indicate cardiac issues that can be identified quickly [31,55,56].

LV mass loss may indicate long-term cardiological damage in these patients [31,57].

To avoid cardiotoxicity, cardiologists and oncologists must collaborate to assess patient risk and provide treatment [58,59].

Cardiotoxicity prophylaxis lowers chemotherapeutic drug cardiotoxicity to prevent cardiovascular events [58,60,61].

An initial cardiological assessment should identify and treat cardiovascular risk factors or pathology and help patients quit smoking, exercising, and dieting [58,62,63]. Echocardiography can track cardiotoxicity risk variables after cancer treatment [58,64,65].

Due to our small sample size and the absence of a long-term follow-up, we could not detect cardiotoxicity in higher-risk individuals. The lack of 3D echocardiography for LVEF assessment before treatment is another study fault. The best LVEF measurement is 3D echocardiography [66,67,68]. First-line echocardiography should assess cancer patients’ heart function [2,9]. When transthoracic echocardiography is neither available nor diagnostic, MRI before chemotherapy for heart function assessment is lacking. Other limitations include the small number of cases due to the small geographic area and the lack of cardiac biomarker screening for ARLVD prediction and cardioprotective therapy reported in other previous studies [69].

## 5. Conclusions

Cardiotoxicity represents a significant complication of chemotherapy, influenced by the specific type and maximum therapeutic dose of the cytostatic agent, as well as the patient’s age, comorbidities, and cardiovascular risk factors.

Regular clinical evaluations and analyses of biological, hemodynamic, vascular, and echocardiographic parameters in patients with AL before and during chemotherapy, particularly in individuals over 60 years and those with cardiovascular risk factors, may enhance long-term outcomes for these patients, even post-chemotherapy.

A thorough clinical examination is essential to identify potential cardiovascular or cerebrovascular conditions prior to initiating any oncological treatment. Identifying and correcting cardiovascular risk factors improves long-term outcomes for patients.

This study indicates that patients exhibiting a higher risk profile present with diminished LVEF and subclinical atherosclerosis, potentially heightening their vulnerability to subsequent cardiotoxicity.

Chemotherapy, particularly in older patients with pre-existing cardiovascular risk factors, induces cardiotoxic effects. Individuals diagnosed with acute leukemia receiving cytostatic treatment, especially anthracyclines, exhibit increased susceptibility to vascular, hemodynamic, and echocardiographic alterations during oncological therapy.

## Figures and Tables

**Table 1:** Demographic data in AL patients with/without CVRFs and controls with CVRFs. SD: standard deviation; AL: acute leukemia; CVRFs: cardiovascular risk factors. * *p* value corresponding to comparison of AL and CVRF versus AL without CVRF patients; ** *p* value corresponding to comparison of AL and CVRF patients versus control with CVRF before chemotherapy treatment.

Participants	AL with CVRFs 26 Patients	AL without CVRFs 19 Patients	*p* Value *	Controls with CVRFs 26 Patients	*p* Value **
Age (19–79) years Mean +/− SD	54.85 ± 16.63	44.00 ± 18.75	*0.047*	58.50 ± 5.21	0.639
Men (%)	16 (61.5%)	10 (52.6%)	0.550	12 (46.2%)	0.202
Women (%)	10 (38.5%)	9 (47.4%)	0.550	14 (53.8%)	0.202

**Table 2:** Assessment of BMI, hemodynamic, and vascular parameters in patients with AL and CVRFs versus AL patients without CVRFs and in patients with AL with CVRFs versus controls with CVRFs before chemotherapy initialization.

BMI, Hemodynamic and Vascular Parameters	AL with CVRFs		AL without CVRFs		*p* Value *	Control with CVRFs		*p* Value **
	Mean ± SD		Mean ± SD			Mean ± SD		
BMI (kg/m^2^)	27.25	3.64	23.09	3.67	**<0.001**	24.57	3.81	0.078
HR (b/min)	79.07	10.53	78.10	10.20	0.758	79.34	17.13	0.864
SBP (mmHg)	126.53	10.84	125.47	9.90	0.738	144.42	20.75	**<0.001**
DBP (mmHg)	76.92	11.66	75.84	11.45	0.759	84.42	12.75	**0.023**
IMT left (mm)	0.78	0.20	0.69	0.12	0.061	0.76	0.15	0.643
IMT right (mm)	0.77	0.22	0.75	0.10	0.683	0.65	0.16	**0.032**
ABI left	1.10	0.08	1.13	0.10	0.275	1.09	0.16	0.553
ABI right	1.09	0.09	1.23	0.37	0.132	1.12	0.25	0.519
PWV (m/s)	7.07	1.15	6.80	0.69	0.376	6.89	1.12	0.742

SD: standard deviation; BMI: body mass index; HR: heart rate; SBP: systolic blood pressure; DBP: diastolic blood pressure; IMT: intima-media thickness; ABI: ankle brachial index; PWV: pulse wave velocity in the aorta. * *p* value corresponding to comparison of AL and CVRF patients versus AL without CVRF patients; ** *p* value corresponding to comparison of AL and CVRF patients versus control with CVRF patients before chemotherapy treatment.

**Table 3:** Echocardiographic parameters of LV systolic and diastolic function before chemotherapy initialization in AL patients with CVRFs versus AL patients without CVRFs and in AL patients with CVRFs versus controls with CVRFs.

Echocardiographic Parameters	AL with CVRFs		AL without CVRFs		*p* Value *	Control with CVRFs		*p* Value **
	Mean ± SD		Mean ± SD			Mean ± SD		
IVS (mm)	10.07	1.52	9.21	1.39	0.057	10.42	1.33	0.567
LVPW (mm)	10.15	1.12	9.00	1.20	**0.002**	10.08	1.30	0.742
LVEDD (mm)	48.50	5.02	45.68	8.37	0.166	49.19	4.17	0.666
LVESD (mm)	27.15	6.89	24.89	6.84	0.282	26.65	5.74	0.812
LVEF (%)	59.26	5.62	64.05	7.43	**0.018**	61.92	6.17	0.117
LVESV (ml)	31.30	12.97	24.63	6.28	**0.045**	32.65	9.35	0.568
LVEDV (ml)	81.42	30.58	76.94	23.86	0.599	78.26	18.96	0.508
LVFS (%)	40.62	12.48	43.62	11.08	0.408	40.80	12.22	0.093
MAPSE (mm)	15.03	4.00	15.26	3.05	0.188	14.50	3.25	0.531

LV—left ventricular; IVS—interventricular septum; PW—posterior wall; FS—fractional shortening; EDD—end-diastolic diameter; ESD—end-systolic diameter; LVEF—LV ejection fraction; EDV—end-diastolic volume; ESV—end-systolic volume, MAPSE—Mitral annular plane systolic excursion. * *p* value corresponding to comparison of AL and CVRF patients versus AL without CVRF patients; ** *p* value corresponding to comparison of AL and CVRF patients versus control with CVRF before chemotherapy treatment patients.

## Data Availability

The data that support the fundings on this study are available from the corresponding author upon reasonable request.
